# Computational Study of the Cation-Modified GSH Peptide Interactions With Perovskite-Type BFO-(111) Membranes Under Aqueous Conditions

**DOI:** 10.1186/s11671-015-0967-3

**Published:** 2015-06-10

**Authors:** Liang Bian, Fa-qin Dong, Mian-xin Song, Jin-bao Xu, Xiao-yan Zhang

**Affiliations:** Key Laboratory of Functional Materials and Devices for Special Environments, Chinese Academy of Sciences, Urumqi, 830011 Xinjiang China; Laboratory for Extreme Conditions Matter Properties, Southwest University of Science and Technology, Mianyang, 621010 Sichuan China

**Keywords:** Density functional theory, Bismuth ferrite, Glutathione, Quantum dot

## Abstract

We elucidated a number of facets regarding glutathione (GSH)-bismuth ferrite (BiFeO_3_, BFO) interactions and reactivity that have previously remained unexplored on a molecular level. In this approach, the cation-modified reduced GSH (or oxidised glutathione (GS·)) formed on the (111)-oriented BiFeO_3_ membrane (namely BFO-(111)) can serve as an efficient quencher, and the luminescence mechanism is explained in aqueous conditions. Notably, we suggest the use of Fe^2+^↓ ion as an electron donor and K^+^ ion as an electron acceptor to exert a “gluing” effect on the glutamic acid (Glu) and glycine (Gly) side chains, producing an exposed sulfhydryl (−SH) configuration. This method may enable the rational design of a convenient platform for biosensors.

## Background

Recently, there has been an increasing interest in the development of glutathione (GSH, γ-glutamyl-cysteinyl-glycine)-capped quantum dots for selective electrochemiluminescence detection due to the ubiquity of the biological micro-electro-mechanical systems (bioMEMS) technique [1]. Due to the general properties of GSH in solution, the immobilisation of active GSH is viewed as an attractive sensor preparation method or technique for the design of new protected interfaces that are able to resist attack by reactive oxygen species (ROS) [2]. In this way, an interesting platform for immobilising GSH has been previously presented. The method follows a general strategy in which an anchoring layer is first deposited by electrografting a cationic salt followed by post-functionalisation of the deposited layer [3]. However, the successful quantum dots used were mainly limited to CdTe-CdS [4], ZnS [5], IrO_2_-hemin-TiO_2_ [6], ZnO-Au [7] etc. Among these quantum dots, some involved complicated interface treatment and had poor photostability. Thus, designing appropriate bioMEMS with photostable and environment-friendly components is still a worthwhile and challenging undertaking.

In solution, superoxide is reported to oxidise GSH to oxided glutathione GSSG, illustrating radical scavenging of the H atom of the -SH group (GSH) by superoxide or transient formation of hydroperoxyl radicals [8]. It is difficult to obtain a pH-redox dual-responsive electric signal due to the effect of ROS quenching in the aqueous environment. One prevailing theory shows that glutathione-complexed iron clusters can create new fluorescence quenching, replacing that of ROS-GSH. Other common metal ions and ROS did not cause interference [9, 10]. Thus, iron-based oxides have potential applications in GSH detection. Currently, the use of bismuth ferrite (BFO) provides an alternative method for GSH monitoring [11]. Compared with semiconductor [12] and organic [13] thin films, the anti-ferromagnetic phase of the BFO membrane has several outstanding features: (i) the creation of one-dimensional conductive channels activated at voltages as low as 1 V demonstrates artificially created ferroelectric vortices in BiFeO_3_ thin films [14]. (ii) The presence of non-toxic, highly stability, highly remnant polarisation (58.9 μC·cm^−2^) and fatigue resistance (up to 4.6 × 10^7^ cycles) [15, 16]. (iii) The divalent iron (Fe^2+^) as reductant that can capture the active GSH groups such as the carboxyl (-COO^−^) and sulfhydryl (−SH) groups [17]. These advantages make the BFO-(111) membrane particularly attractive for biolabeling and biosensing applications.

## Presentation of the Hypothesis

During the course of our investigation on anion-doped BFO, we discovered that the Fe charge disproportionation can be effectively controlled by the oxygen-modified spin polarisation [18]. The increase of the electric field allows a precise determination of the minimally detected electric field due to the iron surface passivation by Fe-anion complexes [19, 20]. Here, we designed the novel GSH-M-BFO (M = K^+^, Rb^+^, Ca^2+^ and Sr^2+^) to immobilise and detect oxidised glutathione (GS·)-reduced glutathione (GSH). The electronic transfer mechanisms were discussed via the DFT technique.

## Testing the Hypothesis

### Methods

GSH is a tripeptide consisting of glutamic acid (Glu), cysteine (Cys) and glycine (Gly) residues. The Glu residue is attached to the Cys residue by its γ-carboxyl group rather than its α-carboxyl group. Usually, a peptide bond is formed between the amino group and the carboxyl group at the α position; however, glutathione forms a peptide bond with the carboxyl group at the γ position of Glu and the amino group of the Cys residue. The plausible adsorbed sites of glutathione would be the carboxyl group at the C terminal, the amide group at the N terminal, and the sulfhydryl (−SH) and carboxyl (-COO^−^) groups of the side chain. To obtain reduced or oxidised GSH under aqueous conditions, we considered the effect of competitive adsorption between the sulfhydryl groups and cationic salts. Therefore, we used surface Fe^2+^ ions to capture the -SH and -COO^−^ groups for designing surface-exposed reduced and oxidised GSH. Additionally, the cationic salts protected the -COO^−^ group to eliminate the effects of free radicals.

We determined the adsorption of cation-modified GSH onto the (111)-oriented BiFeO_3_ membranes (namely BFO-(111)) using 10,000,000 step grand canonical Monte Carlo (GCMC) simulations and 2100 ps isothermal-isobaric (NPT) and canonical ensemble (NVT) molecular dynamics (MD) with a universal force field [21]. The temperature and the pressure of the system were controlled by a Nose-Hoover thermostat and a Berendsen barostat. The calculated diffusion coefficient for all of the species as a diffusion rate on the BFO-(111) surface was a function of both the solution and BFO characteristics, showing a hindered diffusion stage at a simulation time of up to 100 ps, as demonstrated in previous studies. Consequently, the calculated diffusion coefficients after 2000 ps were taken as the representative values for the subsequent analysis [22].

Because the charge transfer of GSH-BFO plays a role in the solvation energy, it is expected to contribute to the final state electronic relaxation of the system after electron ionisation. We calculated the partial densities of states (PDOS) curves and the imaginary part of the dielectric function *ε*_(_*ω*_)_ by a logical extension of the pseudo-potential plane-wave method for the density functional theory (DFT)-generalised gradient approximation (GGA) technique [23, 24]. The Brillouin zone integrations were 3 × 3 ×3 Monkhorst-Pack *k*-point meshes. The calculations of the electronic transitions were performed in the frame of DFT with the Cambridge sequential total energy package (CASTEP) using double numericals with a Perdew Burke Ernzerhof (PBE)-generalised GGA exchange correlation function [25]. A pseudo atomic calculation was performed for the Bi-6p^3^, Fe-3d^6^ and O-2p^4^ valence electron states. The density matrix convergence threshold was set to 10^−4^. A Fermi smearing of 0.005 hartree and a real-space cut-off of 0.45 nm was also used to improve the computational performance.

### Results

**Fig. 1:**
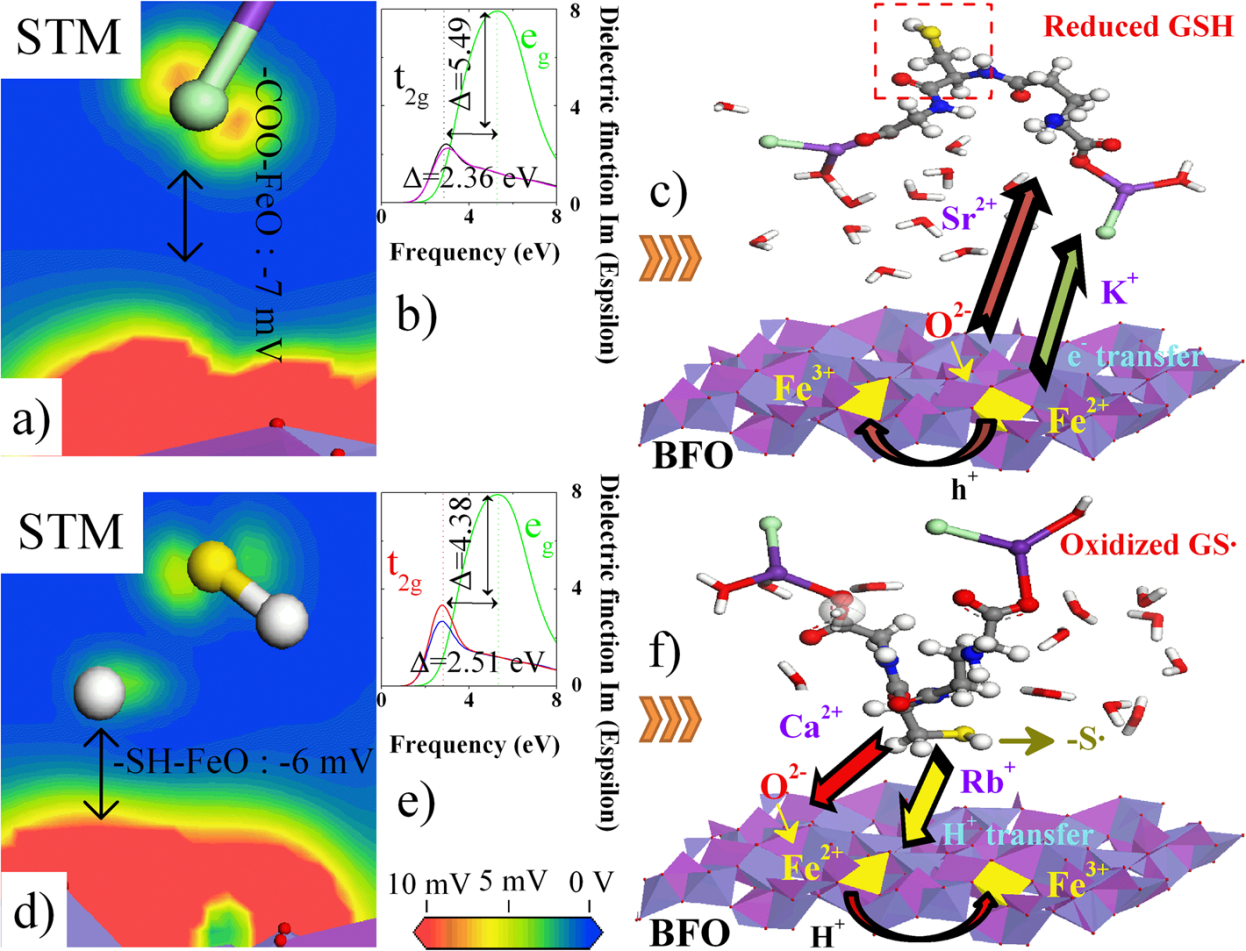
Electron transfer mechanism of **a**–**c** reduced GSH and **d**–**f** oxidised GS·onto BFO-(111) membranes under aqueous conditions. Therein, **a** and **d** reflect the STM images of GSH and GS· onto BFO-(111), respectively. **b** and **e** show the relative dielectric functions. **c** and **f** illustrate the electron transfer processes

The adsorption properties of ions (M: K^+^, Rb^+^, Ca^2+^, Sr^2+^ and Cl^−^) and water (H_2_O) onto the BFO-(111) membranes were initially examined to investigate the effect of compensating ions on the GSH-BFO interaction. Thus, the carboxyl-functionalised and sulfhydryl-functionalised concepts should be first introduced to characterise the ion adsorption modes. For the scanning tunnelling microscopy (STM) results shown in Fig. 1a, d, the lowest voltage at which one can still identify the periodicity of the molecules is 1 mV; lower voltage is prohibited by the BFO substrate. Therefore, the detected voltage decreases ~ −7 mV (K^+^ and Sr^2+^) or ~ −6 mV (Rb^+^ and Ca^2+^), which can be used as the signal feedback of GSH (or GS·)-BFO. Although the signal feedbacks are similar to each other, the electron transfer mechanisms are different due to the effect of the cationic salts. Surprisingly, at 1 mV, we can distinguish between two domains that exhibit the same structure, which may be associated with the two possible adsorption configurations of the GSH and GS· sequences. The binding sites on BFO surfaces are composed of two bidentate sites, i.e. Fe(O)-M-OCO and Fe(O)-S·, formed by terminal and bridging oxygen molecules [26], as shown in Fig. 1c, f.

To reveal this conductive mechanism, we calculated the dielectric constants via the Kramers-Kronig transform [16], see Fig. 1b, e. For anti-ferromagnetic phase BFO, the net magnetic moments correspond to an asymmetry between the spin-up Fe^3+^(↑) and spin-down Fe^2+^(↓) states [17], indicating that the free electrons in the high-energy twofold-degeneracy orbital (*e*_*g*_) can escape from the O-2p^4^↑ orbital to bind directly to the Fe^3+^-3d^6^↑ (or Bi^3+^-6p^3^↑) orbital. The amount of charge carriers lost due to the dropping of quantum wells supports the conclusion that the GSH (or GS·) surface orbital in the electric double layer enhances the angular frequency (*ω*) of the π bond. The left peak (2.6 eV) of the imaginary term *ε* is associated to the triple-degeneracy orbital (t_2g_) from the O-2p^4^↑ to Fe^2+^-3d^5^↓ orbital. Importantly, this chemical response of the BFO-(111) membrane is highly selective toward cation-modified GSH (or GS·). By analysing the O–O spin electron density, we found that the -COO^−^ surface electrons (GSH) preferentially transferred into high potential energy K^+^ and Sr^2+^ orbitals (39.92~40.02 kJ·mol^−1^). These high-energy M-COO electron clouds can capture active Fe-3d (or O-2p) electrons from BFO surfaces, indicating the formation of symmetrical O-O spin states (0.38 and −0.32 hbar). Typically, the electron transfer rates increase in GSH-K^+^-BFO systems due to the effect of the poised potential of the K-4s^2^(4d^0^) and Fe-3d^0^ electron acceptors. One Cl^−^ ion and one bound water molecule of the cyclic K^+^ form a slightly distorted quadrilateral structure that ensures the equal geometric distances in the O-Fe^2+^ and O-K^+^ bonds. Thus, the weakened Fe-O spd3 hybrid orbital reduces the binding force for Fe and O atoms, increasing the geometric distances of the COO^−^-Fe (0.26 and 0.32 nm) and S^−^-O (0.28 and 0.32 nm) bonds compared with the initial O-Fe (~0.2 nm) bonds, as listed in Table 1. This hybrid orbital not only tightly bonds the Glu side chain onto Fe^2+^↓ ions but also pulls some free water into the GSH-BFO interface to enhance the hydrophilic interactions (Gly). In contrast, GSH is oxidised into glutathione disulphide (GS·), when a Rb^+^ (or Ca^2+^) ion modifies the -COO^−^ group. The -S· group induces the polarised O–O bonds to capture approximately one spin electron (−1.29 and −0.99 hbar). These results about the bond selective reactions can be used to explain the differences in the electron transfer mechanism of GSH-GS· detection.

**Table 1:** Diffusion coefficients (×10^−10^ m^2^·s^−1^) and bond lengths (×10^−1^ nm) of the GSH-BFO systems

			KCl	SrCl_2_	RbCl	CaCl_2_
BFO (111)	Diffusion	O	3.55	2	0.5	1.39
		Fe	3.55	2	0.49	1.38
	Bond length	O-Fe	2	2.03	2.04	1.98
GSH	Diffusion	-COO	221.43	119.2	55.16	98.23
		-SH	304.56	122.1	95.5	157.8
		-NH_2_	215.25	123.4	55.5	67.5
	Binding bond		(Cl–Fe) 3.23	(NH_2_–O) 2.56	(SH–Fe) 3.21	(CH_2_–O) 2.77
MCl_n_	Diffusion		167	118.5	65.04	62.03
Water	Diffusion (adsorbed)		341.6	141.4	124.7	89.88
	Binding length	H_2_O–Fe(O)	(Fe) 2.88	(O) 4.4	(Fe) 2.48	(O) 3.22
	Diffusion (free)		792	503	703	260

**Fig. 2:**
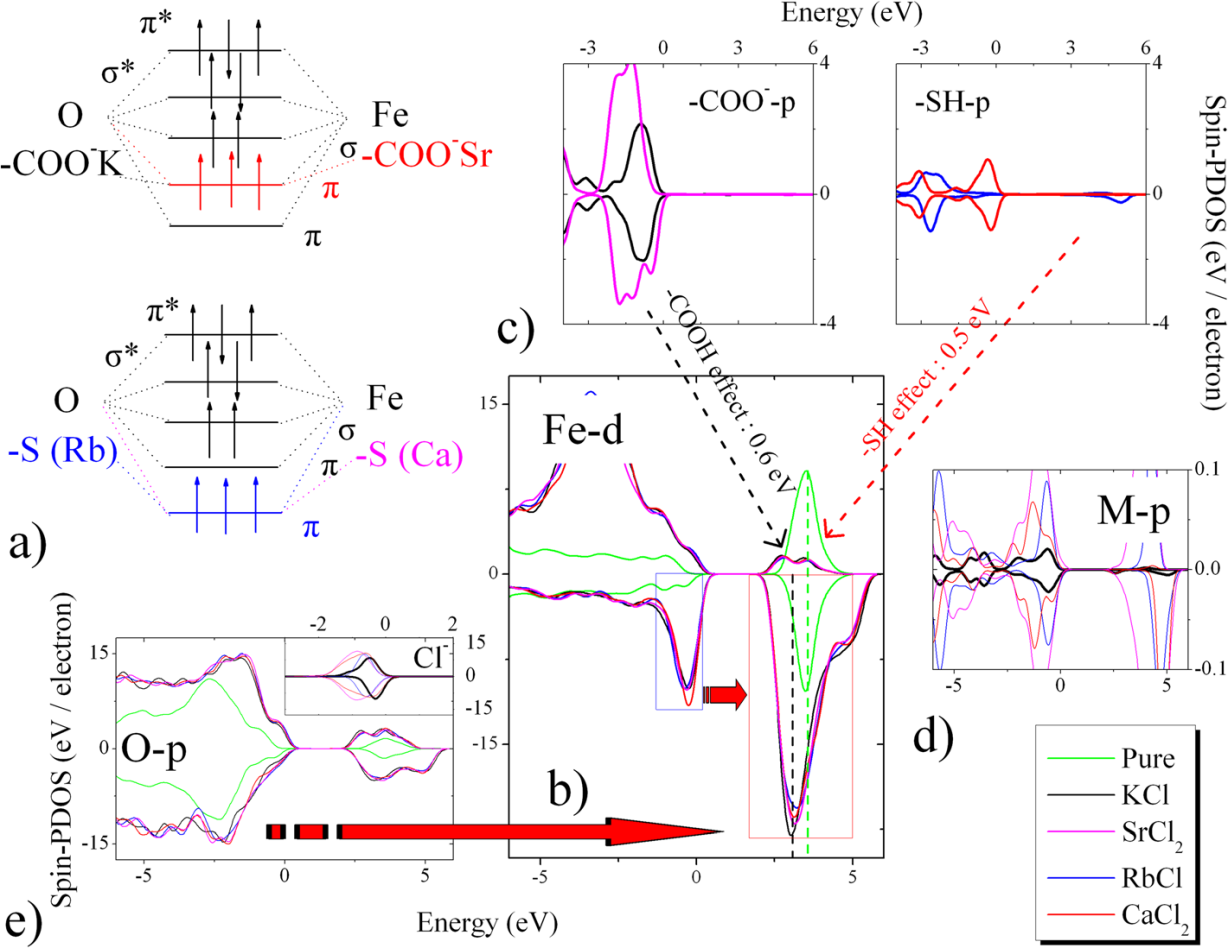
**a** Degenerate states and **b**–**e** pin-PDOSs of cation-modified GSH-BFO systems. Whereas, **b** means the spin-PDOSs of and Fe-3d^5^ orbitals. **c** shows the spin-PDOSs of p orbitals of -COO^−^ and -SH groups. **d**–**e** reflect the spin-PDOSs of p orbitals of cation (M) and O-2p^4^ (Cl-3p^5^), respectively. The σ^*^ and π^*^ mean the anti-bonding orbitals for the σ and π bonding orbitals

We tested the cause of cation-induced electron transfer mechanisms by analysing the orbital change (Fig. 2a). Our unexpected finding triggers a very challenging fundamental question regarding the effect of cation valences on electron transfer mechanisms. For example, the cations cause high orbital degeneracy in the Fe^2+^↓ spin level near the Fermi point (see Fig. 2b). Because the cations attach to the Gly- or Glu-carboxyl, the valence electrons of the p_x_ orbital (-COO^−^) provide a vertical bonding orbital that excites the M-s orbital electrons jumping into the empty d^0^ orbital (Fig. 2c–d). The p_y_ and p_z_ orbitals overlap at the vertical sides, forming two new π bonds. Thus, the Fe-OCO p-d hybridisations are cut off to push up the sixfold-coordinated Fe^3+^↑ states and lead to the inverted band structure (fivefold-coordinated Fe^2+^↓). The formation of a fivefold-coordinated Fe^2+^(O^2−^)-M-COO^−^ t_2g_ or Fe^2+^(O^2−^)-S·-M t_2g_ double electric layer offsets the *e*_*g*_ electron transition in the sixfold-coordinated Fe^3+^(Bi^3+^)-O structure. The increase of localised degree of the fivefold-coordinated Fe–O bond significantly increases the effective electron masses. Taking into account the mediating cation valences, we calculated the surface Mulliken charges to explain the electron transfer rates, as displayed in Table 2. In contrast with the monovalent cations, the divalent cations lose electrons to become positively charged potential wells for trapping O-2p^4^ electrons (BFO). The valence electrons jump into half-full O-2p^4^ levels through sp2 hybridisation. This electron transfer process suggests two charge compensations on M^2+^ (0.68 and 0.23 e) and Cl^−^ (0.08 and 0.54 e). Therefore, the active electrons may skip the positively charged Fe^2+^ potential well and transfer into the negatively charged O^2−^ potential well, as illustrated in Fig. 2e. The relative kinetic energy (29.16~29.2 kJ·mol^−1^) was higher than that (28.71~28.76 kJ·mol^−1^) of the monovalent cation-modified system.

**Table 2:** Mulliken charges (*e*) of BFO, GSH, MCl_n_ and H_2_O

	BFO		GSH			MCl_n_		Water	
	Fe	O	-COO^−^	-N	-S	M	Cl	Adsorbed	Free
Initial	0.85	−0.71	–	–	–	–	–	−0.12	−0.12
KCl	0.81	−0.76	−0.15	−0.56	−0.08	1.12	−0.88	0.1	0.02
SrCl_2_	0.81	−0.76	−0.15	−0.78	−0.07	1.75	−0.81	0.06	0.02
RbCl	0.81	−0.77	−0.13	−0.85	−0.03	1.07	−0.89	0.03	0.05
CaCl_2_	0.75	−0.77	−0.11	−0.81	0.26	1.3	−0.35	−0.08	0.02

**Fig. 3:**
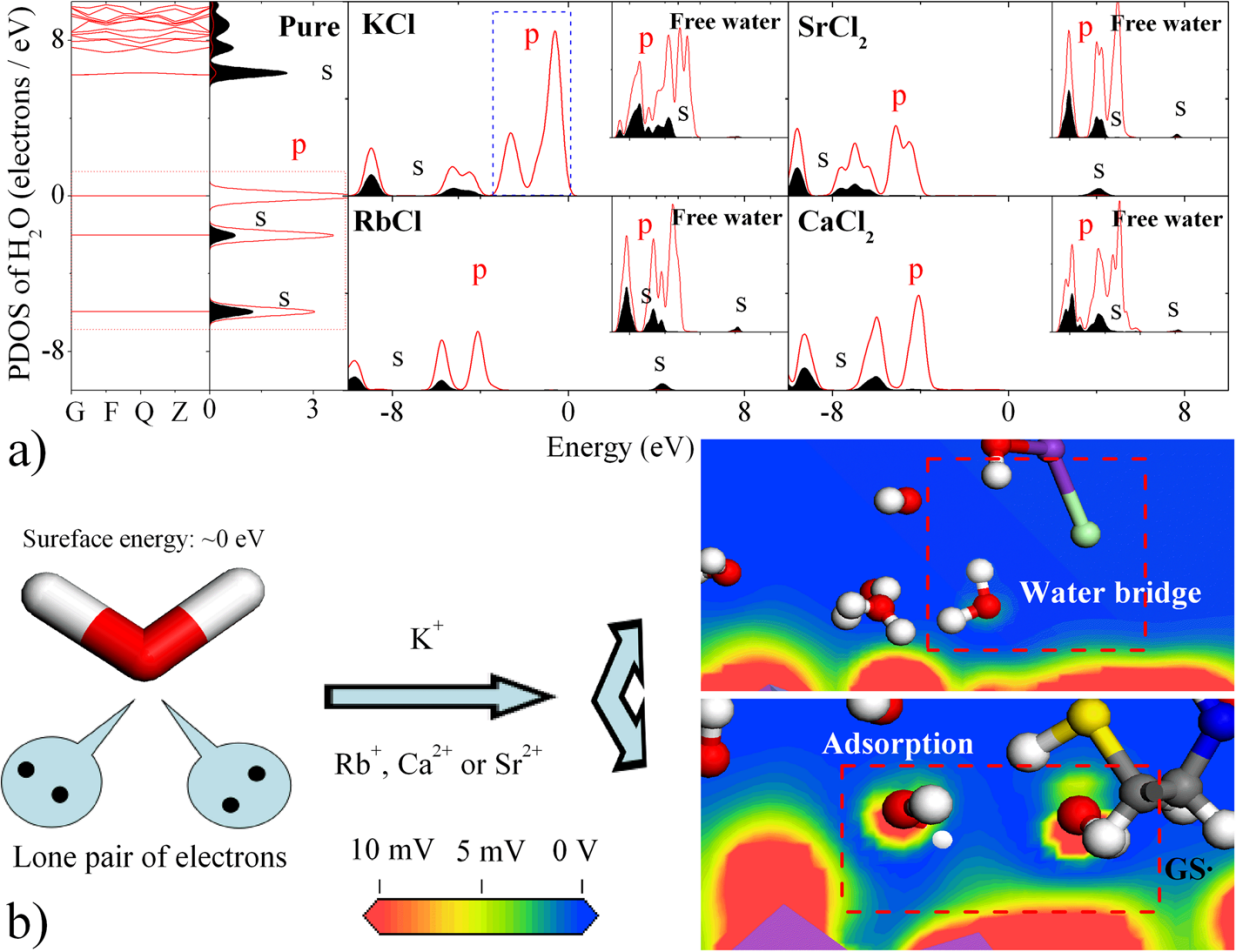
**a** PDOSs of adsorbed and free water molecules in the interlayer of GSH-BFO. **b** shows the illustration of the STM images of water bridge and adsorption interactions

To better understand the binding mechanism of cation-modified GSH at the water-BFO interface, we calculated the electronic structures of the water molecules. Figure 3a demonstrates the presence of a water bridge in the GSH-KCl-BFO system, indicating that two lone pair electrons can escape from the water coordination shell to directly bind to the -S· group. Simultaneously, the lone pair electrons of free water can exchange and compensate among neighbouring free water molecules. The restructured hydrogen bonds enhance the water mobility, showing higher diffusion coefficients than bound water. In contrast with the effect of the -S· group, we found that carboxyl-functionalised K^+^ (Sr^2+^) ions enhance the surface potential (39.92~40.02 kJ·mol^−1^) to create a water bridge, as seen in Fig. 3b, resulting in hydrophilic interactions. As a result, two strong hydrogen bonds coexist in the GSH-BFO interlayer, i.e. the Fe-H_2_O-M-COO^−^ (Gly) and Fe-H_2_O-M-COO^−^ (Glu) bonds in coordination with an inverted horseshoe configuration. The free water participates in the reaction of the Fe–K–COO bond according to the coexistence of free water O-2p^4^ electrons and adsorbed water.

## Implications of the Hypothesis

### Discussion

The ability of biomaterial surfaces to regulate small biomolecular behaviour requires control over the surface chemistry and microstructure. One of the greatest challenges with perovskite-type biomedical micro-devices, such as those recently developed for biosensors and drug delivery, is to improve biocompatibility. Improved biocompatibility may be achieved by modifying the exposed BFO-surface with bioactive peptides. One prevailing theory states that glutathione is an important antioxidant molecule, offsetting the oxidation of sulfhydryl enzyme-SH groups. The expanded cells produce H_2_O_2_ that can be reduced to H_2_O by GSH, and the oxidised GS· is reduced to GSH through the adsorption of GSH reductase (GR) by the liver and erythrocytes. The continued reaction process decreases the detection accuracy. In addition, GSH can exert pro- and anti-oxidant effects depending on the nature of the oxidant and redox status of the iron. An interesting study by Chen [9] brought new insight regarding the functionalised GSH-Fe nanocluster in aqueous solutions, which indicates that there is a significant fluorescence quenching. As a consequence, we designed a new cation-modified GSH-BFO-(111) membrane as a potential biomaterial to replace the traditional CdTe alloy, ZnS, etc.

In the present manuscript, we used five-fold-coordinated Fe^2+^↓ ions as electron donors to immobilise GSH and GS· molecules and used cations to regulate the adsorption status, improving the recognition accuracy of GSH-GS· [12]. The results indicated that the intermediate cations help restrain the conformational behaviour of the peptide backbone, facilitating the structural stability of GSH (or GS·) bound to the BFO-surface. Additionally, we provided a notion to create an exposed -SH configuration using strongly bound K^+^ ions to exert a “gluing” effect on the Glu- and Gly-side-chains. This “gluing” effect is different from the report of oxidised interactions between the -SH and Fe atom. This simple technique can be used to obtain accurate signal feedback of GSH, whose annihilation signal for the H-transfer process is very hard to detect in the oxidation-reduction process. Hence, this fluorescent probe as a sensing platform holds great potential for the accurate detection of GSH-GS· content in human serum samples.

In addition to the application of signal detection, the exposed -SH configuration can be used to eliminate ROS [27]. GSH release requires the use of a sensitive carrier by applying an external electric field [17]. In our approach, the K^+^ ions also enhance the mobility of two strong hydrogen bonds (COO–H_2_O and H_2_O–Fe(O)). The water bridge interaction suggests that the fivefold-coordinated Fe atom is surrounded by surface bridging oxygen atoms and is bound to a water molecule. This small probability may induce a controllable desorption of the GSH-sequence from a BFO-(111) membrane. Interestingly, this GSH content can be regulated by changing the BFO surface current to control the adsorption-desorption reaction. For example, the charging on the Fe–O bond provides effective electrons to enhance the transfer rate of lone pair electrons of free H_2_O molecules, improving the localised state of the H_2_O–K–COO bond. The desorption process can be achieved in the discharge process. In summary, this “switching” effect can distinguish the GSH and GS· molecules, providing new potential applications in directional drug release and content detection.

### Conclusions

Because the presence of GSH-GS· peptides on a surface has been shown to produce adhesive substrates, immobilising GSH on a surface is significant to the long-term success of a cell-surface interface. Here, we suggest a simple and fast detection technique for GSH and GS· molecules via the DFT method, and our results support the notion of controlling the electron transfer mechanism using cations. By taking advantage of the highly sensitive GSH and GS·, we have also demonstrated the ability to monitor GSH adsorption using a cation-modified -COO^−^ group. These findings are important not only for providing a sensor platform useful for quantum dot studies but also for enabling promising applications in targeted drug delivery.

Well-defined surfaces with highly controlled molecular and micro-structural architectures have considerable potential in biomaterial investigations. Further investigations should focus on the dynamic responses and electron transfer mechanisms of the BFO-M-(H_2_O)-GSH(or GSSG)-M-(H_2_O)-ROS system in the localised human micro-environment, such as the GSH-dependent ROS response dynamics and hemocompatibility.

## Abbreviations

BFO: bismuth ferrite, BiFeO_3_
BFO-(111): (111)-oriented BiFeO_3_ membrane
bioMEMS: biological micro-electro-mechanical systems
C: carbon
CASTEP: Cambridge sequential total energy package
-COO^−^: carboxyl
Cys: cysteine
DFT: density functional theory
*e*_*g*_: twofold-degeneracy orbital
GCMC: grand canonical Monte Carlo
GGA: generalised gradient approximation
Glu: glutamic acid
Gly: glycine
GR: GSH reductase
GS·: oxidised glutathione
GSH: reduced glutathione, γ-glutamyl-cysteinyl-glycine
MD: molecular dynamics
N: nitrogen
NPT: isothermal-isobaric
NVT: canonical ensemble
PBE: Perdew Burke Ernzerhof
PDOS: partial densities of states
ROS: reactive oxygen species
-SH: sulfhydryl
STM: scanning tunnelling microscopy
t_2g_: triple-degeneracy orbital
*ε*: dielectric function
